# Portal Vein Thrombosis in Second Trimester of Pregnancy

**DOI:** 10.3390/jcm14051713

**Published:** 2025-03-04

**Authors:** Adriana Gregušová, Martina Slováčiková, Katarína Gazdíková, Miroslava Dobrotová, Michaela Jezberová, Miroslav Žigrai

**Affiliations:** 1 1st Department of Internal Medicine, Slovak Medical University & University Hospital Bratislava, 82606 Bratislava, Slovakia; adriana.gregusova@kr.unb.sk (A.G.); miroslav.zigrai@kr.unb.sk (M.Ž.); 2 Department of General Practice Medicine, Medical Faculty, Slovak Medical University, 82606 Bratislava, Slovakia; 3 Department of Hematology & Transfusions, Jessenius Medical Faculty, Comenius University, 03601 Martin, Slovakia; 4 The Dr. Magnet Kramáre, Centre for Magnetic Resonance Imaging, 83305 Bratislava, Slovakia

**Keywords:** portal vein thrombosis, pregnancy, non-obstetric maternal morbidity, maternal outcome

## Abstract

**Background:** Acute portal vein thrombosis can be asymptomatic or may present with non-specific symptoms, making awareness and vigilance crucial among pregnant patients. The management of portal vein thrombosis (PVT) diagnosed during pregnancy is not well defined, as most existing data relate to cases diagnosed before pregnancy. Symptoms can resemble other pregnancy-related conditions, posing a challenge for clinicians. PVT during pregnancy can be effectively and safely managed with anticoagulation therapy. However, the potential for complications necessitates a multidisciplinary approach. This article outlines the case of PVT in a 39-year-old woman in the 14th week of gestation who was admitted through the emergency department due to an acute onset of abdominal pain predominantly in the epigastric and right hypochondriac regions. **Methods:** Abdominal ultrasonography revealed PVT, and treatment with low-molecular-weight heparins was commenced. Further investigation excluded any form of thrombophilic state. **Results:** The patient continued an adjusted dosage of thrombolytics after discharge until the end of her pregnancy and was reinstated for thromboprophylaxis following a caesarean section. **Conclusions:** A thorough diagnosis is vital for any abdominal pain in pregnancy. A personalised approach is essential for effectively managing PVT, highlighting the need for early detection and comprehensive care to optimise outcomes for both the mother and the offspring.

## 1. Introduction and Clinical Significance

Portal vein thrombosis (PVT) is a rare manifestation of thromboembolic events in the abdominal area. It poses a significant clinical concern during pregnancy due to its potential complications and the unique physiological changes that occur during this period. The incidence of PVT in pregnant women is affected by various factors, including the hypercoagulable state associated with pregnancy, pre-existing medical conditions, and the presence of supplementary risk factors.

Regardless of its low prevalence, it represents a life-threatening condition [1,2]. Pregnancy is physiologically associated with changes in haemostasis, including an increase in most clotting factors, a decrease in natural anticoagulants, and a reduction in fibrinolytic activity. These changes result in a state of hypercoagulability due to hormonal changes and lead to an increase in the risk of thromboembolism. The most severe thromboembolic events include stroke, pulmonary thromboembolism, deep vein thrombosis in different areas of the body, and thrombotic events occurring in the abdomen and pelvis.

Etiologically, the causes—particularly in parturient cases—are often multifactorial. The reported prevalence of thromboembolic events during pregnancy is 0.5 to 2.0 per 1000 pregnancies, whereas approximately 75–80% of embolic events in pregnancy are venous [3,4,5,6,7,8,9]. Management and treatment are individualised due to the specifics of each case, including comorbidities, duration of the pregnancy, and foetal condition.

We present a unique case of a parturient woman with acute portal vein thrombosis at the 14th week of gestation without any previous indications of blood or coagulation disorders.

## 2. Case Presentation

A 39-year-old pregnant woman was admitted to the hospital as an emergency due to colic-like abdominal pain that had persisted for three days in the epigastric region, radiating to the right side beneath the rib cage.

The diagnosis of these issues led to her spending one day in the surgical clinic, where antispasmodic treatment resulted in a partial improvement in her condition, facilitating her discharge. This was her fifth pregnancy, two of which had ended in miscarriages during the eighth and ninth weeks. Due to cholecystolithiasis, she had previously undergone an elective cholecystectomy without complications. The patient had used hormonal contraception in the past without complications or side effects.

Upon admission to the hospital, she was not receiving any pharmacotherapy and exhibited no comorbidities. The patient was a non-smoker, refrained from alcohol consumption, had a normal physique, led a healthy lifestyle, and reported no chronic symptoms in her medical history. There was no evidence of abdominal injuries, pancreatitis, myeloproliferative diseases, or thromboembolism, and before her admission, there were no signs of acute infection or other potential causes of thromboembolic events.

The patient had previously been evaluated for both congenital and acquired thrombophilia, which was not substantiated with regard to her mother’s portal vein thrombosis and superior mesenteric artery thrombosis, necessitating intestinal resection.

Upon admission, she was haemodynamically stable and afebrile. A physical examination revealed tenderness in the epigastric region and beneath the right costal margin.

### 2.1. Initial Laboratory Analysis

Upon laboratory evaluation following admission, we observed elevated serum D-dimer levels (1.26 mg/L) and normocytic, normochromic anaemia at a moderately severe level (98 g/L). The thrombophilia assessment indicated that antithrombin III, protein C, protein S, factor VII, and factor VIII were within comparable ranges; anti-cardiolipin antibodies IgG and IgM were negative, and the JAK2 mutation was absent. The serum homocysteine level remained within a physiological range. Infection and autoimmune diseases were ruled out as aetiological factors.

### 2.2. Imaging Methods

**Figure 1:**
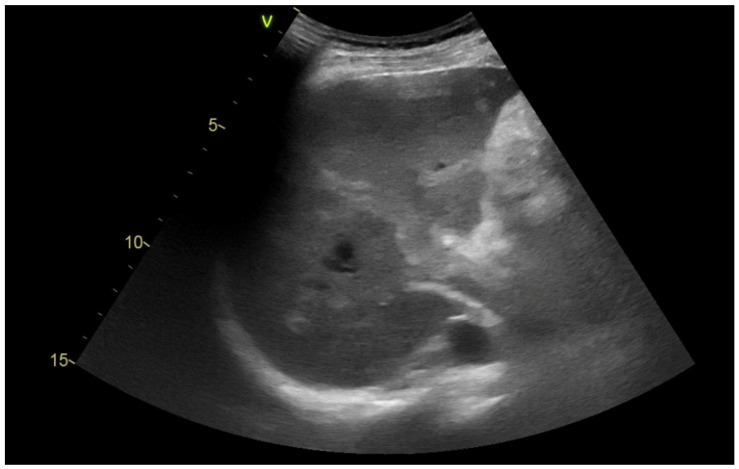
The lumen of the portal vein is dilated and filled with hyperechoic content. This thrombus spreads distally to the portal branch in the right lobe of the liver and extends proximally to the distal part of the superior mesenteric vein.

An abdominal ultrasound conducted post-admission revealed a homogeneous, hyperechoic mass within the lumen of the dilated portal vein (16 mm), encroaching upon its branches in the right lobe of the liver and extending to the superior mesenteric vein, as evidenced by recent venous thrombosis (Figure 1).

**Figure 2:**
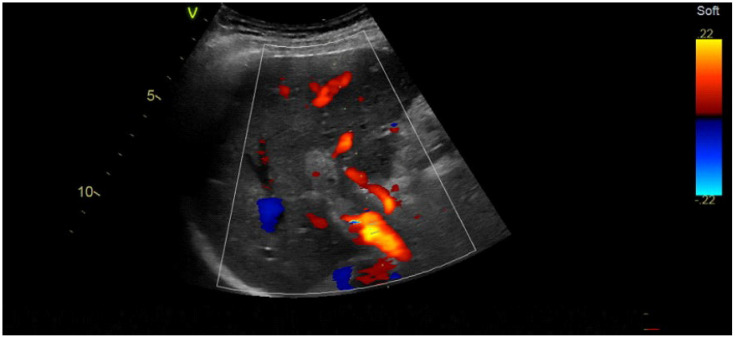
Doppler ultrasound imaging of the portal vein thrombosis.

**Figure 3:**
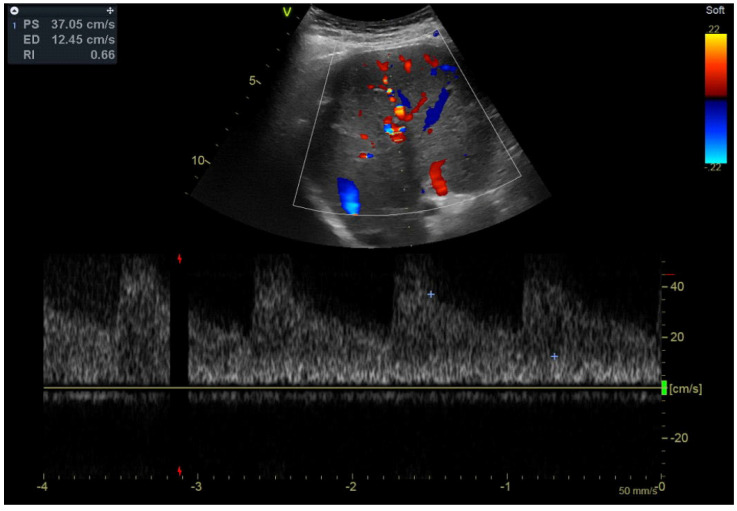
Doppler ultrasound of the hepatic artery with an increased compensatory flow. *Source*: AG.

**Figure 4:**
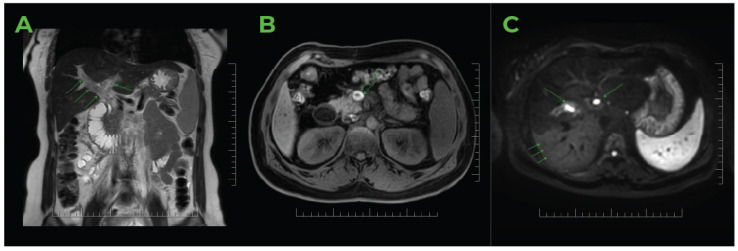
MRI study of the abdomen of pregnant women in the 14th week of gestation. (**A**) The coronal plane T2 demonstrated an abnormal hyperintense signal in the dilated lumen of the portal vein, affecting the branches to the right and left liver lobes. The absence of a flow void signal indicates vein thrombosis, with accompanying findings of splenomegaly. (**B**) The axial plane T1 exhibited a pathological hyperintense signal in the enlarged lumen of the superior mesenteric vein, confirming the presence of a thrombus. (**C**) The axial plane DWI highlighted the thrombus in the branches of the portal vein in the liver (S8/S5, S2/3), as well as a difference in signal intensity within the parenchyma of the right lobe (S7/S6), confirming uneven vascular perfusion. *Source*: MJ.

The colour Doppler ultrasound test corroborated the findings (Figure 2). The hepatic artery showed enhanced compensatory flow (Figure 3), and no portal venous collateral circulation was observed (Figure 3). An obstetric ultrasound confirmed the presence of a viable foetus at 14 weeks of gestation. The assessment was further enhanced by magnetic resonance imaging (Figure 4), which confirmed thrombosis of the superior mesenteric vein, the portal vein, and its branches in the right lobe of the liver. No signs of cholestasis were noted.

### 2.3. Therapy

**Figure 5:**
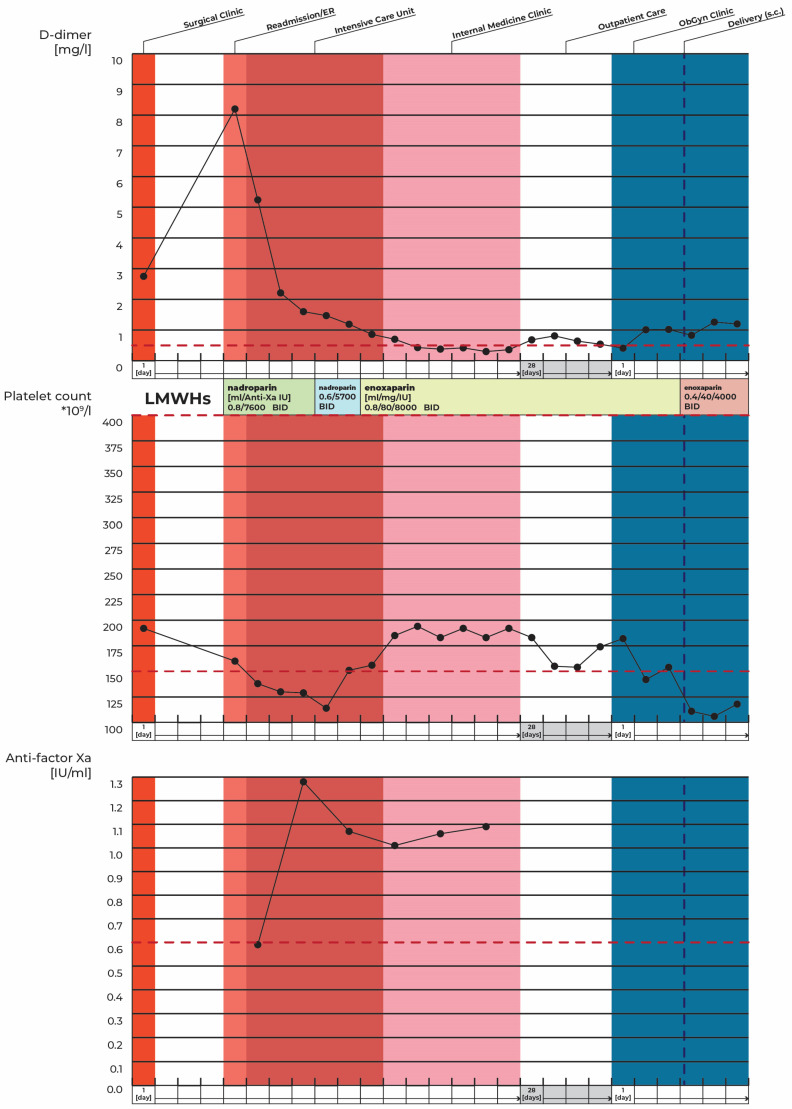
Selected laboratory parameters and anticoagulation therapy regimens over time. The red dashed line represents the reference value for the parameter.

Regarding the patient’s acute portal vein thrombosis, we commenced treatment with the anticoagulant nadroparin, following the dosage guidelines while continuously monitoring anti-Xa activity to attain therapeutic levels (Figure 5).

Due to a decrease in thrombocyte levels, we switched to enoxaparin at its full anticoagulant dosage, which was subsequently accompanied by a recovery of thrombocyte counts (Figure 5).

Concurrently, she received antispasmodic medication, resulting in favourable outcomes. Regarding the patient’s pregnancy, there was no need for parenteral or local thrombolysis or thrombectomy. After several days of treatment, the clinical symptoms were reduced. The patient showed no signs of haemorrhage.

During her hospitalisation, an obstetrician examined her repeatedly and confirmed the ongoing viability of the foetus. Repeated colour Doppler ultrasounds revealed the progressive recanalisation of the portal venous system.

Furthermore, the patient’s pregnancy progressed without complications, and she was discharged at 16 weeks of gestation in a clinically stable condition, with a reduced LMWH anticoagulant regimen until delivery. Following the complete recanalisation of the portal venous system, subsequent laboratory and ultrasound monitoring took place at the hepatology clinic.

At 36 weeks of gestation, the patient was admitted to the gynaecology and obstetrics clinic for a scheduled delivery. The procedure was performed via caesarean section due to the foetal breech position and indications of intrauterine growth restriction, and it proceeded without complications. Following the stabilisation of haemostasis post-delivery, the previously reduced LMWH anticoagulant therapy was reinstated.

### 2.4. Details of Further Therapeutic Considerations Due to Family History

As previously mentioned, the patient’s mother experienced several thromboembolic events in the abdominal cavity. Following the initial event of portal vein thrombosis, she was transitioned from six months of LMWH to parenteral sulodexide, which was subsequently followed by oral sulodexide in combination with ASA.

During this treatment, she experienced another thrombosis of the mesenteric artery. Following a conservative approach that included endovascular surgery, she transitioned once again from LMWH to sulodexide.

Within a year, she experienced another thrombosis of the mesenteric artery, requiring significant intestinal resection. These personal experiences of the patient’s mother impacted her decisions regarding further treatment. Now a mother of three, she aimed to minimise all potential risks and chose to continue with the subcutaneous administration of LMWH.

### 2.5. During and Following Hospitalisation in an Internal Medicine Clinic

The patient was evaluated by a haematologist, who, considering both personal and familial histories, assessed platelet haemostasis, revealing platelet aggregation consistent with sticky platelets syndrome:

(a) Thrombocyte aggregation induced by ADP: Aggreg.Tr-ADP-10 µM: 88%, Aggreg.Tr-ADP-2.34 µM: 86%, Aggreg.Tr-ADP-1.17 µM: 79%, Aggreg.Tr-ADP-0.58 µM: 24%.
(b) Thrombocyte aggregation induced by epinephrine: Aggreg. Tr-EPI-300 µM: 90%, Aggreg.Tr-EPI-11.0 µM: 88%, Aggreg.Tr-EPI-1.1 µM: 77%, Aggreg.Tr-EPI-0.55 µM: 67%. Regulatory assessment for antiphospholipid syndrome: PTT-LT control: 33.6 s, PTT-LT patient: 36.1 s, PTT-LT ratio: 1.07. PTT-LA control: 32.7 s, PTT-LA patient: 36.0 s, PTT-LA ratio: 1.10. Kaolin clotting time: 0.91, dRVVT patient screening: 33.1 s, dRVVT screen control: 34.4 s, dRVVT screen ratio: 0.96 TTI 1:50 ratio: 1.35 s, TTI 1:500 ratio: 1.53 Anti-ß2 GPI-IgG: 1.3 GU/mL, Anti-ß2 GPI-IgM: 1.3 MU/mL.

The presence of antiphospholipid antibodies was not confirmed. The plasma levels of fibrinogen during coagulation were within comparable limits.

The supplementary haematological analysis revealed increased platelet aggregation, meeting the sticky platelet syndrome type I diagnostic criteria.

This condition can cause hypercoagulation, which may lead to thrombosis in both venous and arterial systems. Current examinations do not clarify whether it is congenital or acquired; nevertheless, this distinction does not impact the effectiveness of the treatment approach.

The remaining thrombophilia screening parameters assessed did not indicate the presence of an additional severe thrombogenic condition.

The patient was advised to follow a general thrombosis prevention regimen, which includes maintaining physical activity, avoiding injuries and illnesses, and ensuring adequate hydration. Considering the risks, family history, and overall condition, the patient was recommended to administer LMWH (nadroparin 3700 IU/(anti-Xa)/0.4 mL once daily).

### 2.6. Neonatal Outcome and Further Condition of the Offspring

Due to the PVT, IUGR, and the baby’s breech position, an elective caesarean section was performed at 36 weeks of gestation. With Apgar scores of 7, 9, and 9, the boy weighed 1560 g and measured 40 cm long. The placenta showed no obvious pathology and weighed 400 g. The immature neonate encountered challenges with postnatal adaptation, requiring non-invasive pulmonary ventilation. Penile angulation and proximal penile hypospadias were observed. He spent 48 h in the NICU, with a total hospital stay of three weeks in the neonatology ward. During his first 18 months, he encountered only common childhood illnesses and had no health issues; his psychomotor development was adequate and free from pathologies. He has already undergone phase 1 of penile reconstruction surgery without complications.

### 2.7. Further Outpatients’ Care

**Figure 6:**
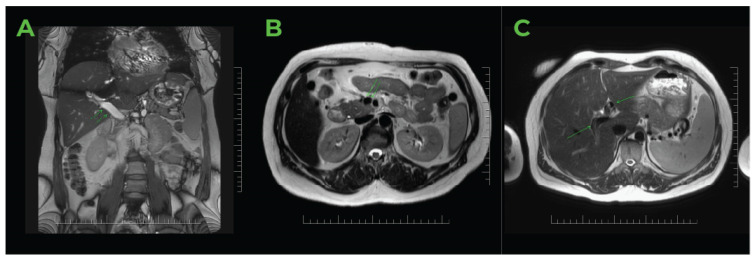
Control abdominal MRI study 15 months after delivery. (**A**) The coronal plane BTFE sequence showed renewed flow signals in the previously thrombosed portal vein and its branches within the liver, confirming complete recanalisation. (**B**,**C**) The axial plane T2 confirmed the normalisation of the lumen width and the flow signals of the superior mesenteric vein along with the branches of the portal vein in the liver. *Source*: MJ.

Assessments of the clinical condition were ongoing, and serum D-dimer levels were closely monitored through titration. One year postpartum, the patient is stable and shows no signs of recurrent thrombosis or complications from post-thrombotic syndrome (Figure 6).

## 3. Discussion

Deep venous thrombosis affecting the portal vein and its extrahepatic components, including the superior mesenteric and splenic veins, is exceedingly rare during pregnancy and is primarily noted in the literature as case reports.

Non-cirrhotic, non-tumoral PVT is the second most frequent cause of portal hypertension worldwide. General thrombophilic factors can be identified in approximately 60% of patients. PVT may present as an acute process; however, the acute episode is often asymptomatic or paucisymptomatic, leading to misdiagnosis of portal vein thrombosis until complications arise from portal hypertension, such as portal biliopathy or variceal bleeding [10].

In patients with recurrent thrombosis, thrombophilia is frequently observed, often indicating factor III deficiency, antiphospholipid syndrome, protein C or S deficiency, or factor V Leiden or factor II deficiency. Further assessments to determine the presence of a prothrombotic state include the JAK2 mutation, with or without myeloproliferative disorders, prothrombin gene mutation, antithrombin deficiency, paroxysmal nocturnal haemoglobinuria, and vasculitis, which encompasses Behcet’s disease, methylene tetrahydrofolate reductase deficiency, and genetic mutation of plasminogen activator inhibitor-1.

Hypercoagulation during pregnancy increases the risk of deep vein thrombosis, including portal vein thrombosis [11,12,13]. The use of hormonal contraception, especially among women aged 35 and older, may further exacerbate this condition [14].

Local and systemic factors play a significant role in the pathophysiology of portal vein thrombosis; however, other risk factors also exist.

The published case studies highlight the following causes of portal vein thrombosis: sticky platelet syndrome type 2, thalassaemia [15], congenital hepatic fibrosis [16], omphalitis [17], hepatic haemangioma, protein S deficiency [18], hepatocellular carcinoma, thrombophilia [19], antiphospholipid syndrome [20], and COVID-19 infection [21]. It has also been observed in patients after artificial insemination [22]. Sticky platelet syndrome (SPS) is characterised by the hyperaggregability of platelets upon exposure to low levels of adenosine diphosphate (ADP) and/or adrenaline (EPI). Clinical signs include unexplained arterial and venous thrombosis, which occur in stressful circumstances and often relapse despite adequate anticoagulant therapy. A causal link has been established between SPS and miscarriages. SPS is classified into type I (enhanced aggregation following EPI and ADP), type II (enhanced aggregation following EPI), and type III (enhanced aggregation following ADP). Type II SPS is the most prevalent variant.

The prevalence in the population remains indeterminate since the only research conducted has involved patients experiencing a thromboembolic event. Similarly, we cannot ascertain the prevalence within this population segment, as it primarily comprises small cohorts of individuals with unexplained symptoms.

SPS is an inherited disorder characterised by an autosomal dominant, likely polygenic, inheritance pattern. Although its phenotypic traits are well established, the exact genetic origin remains unknown. The pathophysiology is believed to involve a defect in platelet membrane glycoproteins or intracellular signalling pathways associated with thrombocyte activation and aggregation.

The process of thrombocyte aggregation involves the interaction of numerous proteins. Genetic mutations may be the underlying cause of SPS. Prominent candidates include GPIa/IIa, GPIb/IX/V, GPVI, GPIIb/IIIa, PGE2 receptor, thrombin receptors, α2-adrenergic receptors, GAS6, and PEAR1 protein. A particular genetic defect responsible for platelet hyperaggregability has yet to be identified.

SPS is a condition identified in individuals with arterial and venous thrombosis. Thrombosis frequently occurs in common sites. Venous thrombosis typically involves deep vein thrombosis in the lower limbs, while arterial instances pertain to the coronary and cerebral arteries. Thrombosis has also been reported in unexpected locations where SPS predominates.

The initial thrombotic event occurs in the young, specifically during the third or fourth decade of life, among patients with limited acquired thrombotic risk factors. Currently, SPS is associated with intrauterine growth restriction of the foetus, abnormalities in placental vascularity, and miscarriages. A defining characteristic of SPS is the advancement or recurrence of thrombosis despite adequate anticoagulant therapy (using heparin or coumarin).

The favourable family history of thromboembolic events in consanguineous individuals illustrates the genetic basis of SPS. Additional factors, including oral contraceptives, smoking, and stressful situations, influence the risk of thrombosis linked to SPS [23].

Research has shown the effectiveness of antiaggregatory treatment therapeutically and as a preventative measure for thrombosis in cases of SPS. Despite considerable variability in antiaggregatory therapies, acetylsalicylic acid (ASA) remains the primary treatment, whether used alone or in combination with other agents. At the same time, the risk of recurrent thrombosis is typically minimal [24,25]. A low dose of ASA (80–100 mg) is an effective and safe therapeutic option for most individuals.

In cases of ASA resistance or contraindications, alternative antiplatelet agents may be utilised, with ADP inhibitors being the most suitable and effective.

Managing individuals with SPS and other thrombophilia conditions can be complex. Treatment often requires a tailored approach, frequently involving a mix of antiaggregatory therapy with low-molecular-weight heparin or a vitamin K antagonist, depending on the specific issues at hand. There are no universal guidelines for treatment; it is wholly individualised.

The clinical presentation of PVT varies based on the speed of thrombus formation, location, portal vein obstruction degree, and extrahepatic branch dilation.

Acute portal vein thrombosis may present suddenly with abdominal pain, which may be accompanied by fever or digestive issues. In severe cases, it can lead to ischaemia, including mesenteric ischaemia. If not treated quickly, this condition may progress to intestinal perforation, peritonitis, shock, and multi-organ failure. Liver enzyme levels might be within normal range or significantly elevated. Patients with portal biliopathy often show increased ALP and GGT levels [26]. If clinical suspicion arises, abdominal ultrasonography and hepatic blood flow evaluations should be performed promptly, as these techniques are highly sensitive and specific for diagnosis. CT and MRI scans also provide essential insights.

MRI examinations are recommended during pregnancy due to their non-invasive characteristics. Unlike CT scans, MRI does not utilise ionising radiation and provides superior soft-tissue specificity. It operates without intravenous contrast media, using various sequences to identify thrombosis in venous lumens while imaging nearby organs and structures and any associated ischaemic damage [27,28,29].

The prognosis for patients depends on the underlying condition and long-term management; however, the prognosis is favourable for those on anticoagulant medication, with over 70% of patients surviving beyond 10 years [30].

The management of portal venous thrombosis and addressing the underlying illness varies depending on whether the condition is acute or chronic.

The management of acute portal venous thrombosis relies on promptly administering anticoagulant medication. Following expert recommendations, the latter aims for rapid revascularisation and eradicating and preventing thrombus propagation [31,32,33]. Complete recanalisation is achieved in approximately 30–40% of all cases. The duration of anticoagulant therapy depends on the severity of the thrombosis, the level of occlusion, the seriousness of the underlying condition, and its duration. The provision may extend for six months or more.

Approximately 40% of patients tend to develop portal cavernoma [11].

Thrombolysis is rarely required; however, case studies effectively illustrate local thrombolysis in the early stages of portal venous thrombosis.

Long-term anticoagulant therapy and proper prevention of sequelae, particularly the anticipated haemorrhage from oesophageal varices, are essential for effectively treating portal vein thrombosis, even during pregnancy. Given the risk of haemorrhage, even in elective caesarean deliveries, as in our patient’s case, continuous monitoring of coagulation parameters is crucial [19].

Consequently, a multidisciplinary approach and collaboration amongst the hepatologist, gastroenterologist, haematologist, and gynaecologist/obstetrician are vital for effectively managing acute portal vein thrombosis during pregnancy, necessitating precise diagnosis and treatment.

## 4. Conclusions

Acute portal vein thrombosis is an uncommon aetiology of abdominal pain that should be prioritised in patients with known risk factors or a family history of thrombosis.

Pregnancy increases personal risk factors for hypercoagulation. Timely findings from an ultrasonographic assessment of hepatic blood flow are essential for the early initiation of anticoagulant therapy and for preventing severe acute and chronic consequences.

Owing to its accessibility and cost-effectiveness, ultrasonography serves as an effective means for diagnosing and monitoring portal vein thrombosis, particularly during pregnancy.

## Data Availability

Data sharing does not apply to this review as no new data were generated.
